# Insertion of Calcium-Permeable AMPA Receptors during Epileptiform Activity In Vitro Modulates Excitability of Principal Neurons in the Rat Entorhinal Cortex

**DOI:** 10.3390/ijms222212174

**Published:** 2021-11-10

**Authors:** Dmitry V. Amakhin, Elena B. Soboleva, Anton V. Chizhov, Aleksey V. Zaitsev

**Affiliations:** 1 Sechenov Institute of Evolutionary Physiology and Biochemistry, Toreza Prospekt 44, 194223 Saint Petersburg, Russia; dmitry.amakhin@gmail.com (D.V.A.); soboleva.elena.1707@gmail.com (E.B.S.); anton.chizhov@mail.ioffe.ru (A.V.C.); 2 Ioffe Institute, Russian Academy of Sciences, Polytekhnicheskaya 26, 194021 Saint Petersburg, Russia

**Keywords:** epilepsy, synaptic plasticity, NMDA receptor, excitatory postsynaptic current, IEM-1460, patch-clamp, brain slice

## Abstract

Epileptic activity leads to rapid insertion of calcium-permeable α-amino-3-hydroxy-5-methyl-4-isoxazolepropionic acid receptors (CP-AMPARs) into the synapses of cortical and hippocampal glutamatergic neurons, which generally do not express them. The physiological significance of this process is not yet fully understood; however, it is usually assumed to be a pathological process that augments epileptic activity. Using whole-cell patch-clamp recordings in rat entorhinal cortex slices, we demonstrate that the timing of epileptiform discharges, induced by 4-aminopyridine and gabazine, is determined by the shunting effect of Ca^2+^-dependent slow conductance, mediated predominantly by K^+^-channels. The blockade of CP-AMPARs by IEM-1460 eliminates this extra conductance and consequently increases the rate of discharge generation. The blockade of NMDARs reduced the additional conductance to a lesser extent than the blockade of CP-AMPARs, indicating that CP-AMPARs are a more significant source of intracellular Ca^2+^. The study’s main findings were implemented in a mathematical model, which reproduces the shunting effect of activity-dependent conductance on the generation of discharges. The obtained results suggest that the expression of CP-AMPARs in principal neurons reduces the discharge generation rate and may be considered as a protective mechanism.

## 1. Introduction

The α-amino-3-hydroxy-5-methyl-4-isoxazolepropionic acid receptors (AMPARs) mediate most of the fast excitatory synaptic transmission in the central nervous system. AMPARs are tetrameric complexes of four subunits (GluA1-GluA4) [1]. The subunit composition of AMPARs determines their functional properties, trafficking, synaptic localization, interaction with intracellular proteins, and Ca^2+^ permeability [2,3]. For example, a lack of GluA2 subunit or the presence of an unedited GluA2 subunit in the receptor complex renders the AMPAR to be permeable to calcium ions [4]. In the adult brain, most glutamatergic neurons express only the calcium-impermeable AMPARs [2], whereas most GABAergic neurons typically express the calcium-permeable (CP) AMPARs [5,6].

The process of transition from self-terminating seizures to status epilepticus (SE) is still not fully understood. Two main mechanisms were characterized. The first one is the decrease of the efficiency of GABAergic inhibition, which is either due to the increase of chloride ion concentration inside the cells [7] or a reduction of GABAa receptor-mediated conductance. The latter can be achieved through several mechanisms, such as enhanced internalization of GABAa receptors [8,9], or the decrease in neurosteroid levels, as shown in humans [10,11] and tested in rats [12]. The second major mechanism is the enhancement of excitatory synaptic transmission. Epileptic activity in vivo and in vitro induces significant potentiation of glutamatergic synapses in the hippocampus [13,14,15,16,17] and the entorhinal cortex [18]. Seizure-induced potentiation is accompanied by the insertion of CP-AMPARs into the synapses of glutamatergic neurons [15,16,18]. It is suggested that the potentiation of excitatory synapses can be one of the mechanisms of transition from seizures to SE [19,20]. The antiepileptic effect of CP-AMPAR antagonists in vivo also points to the pathological role of CP-AMPARs [21,22,23].

In contrast, our previous results indicate that incorporation of CP-AMPARs might have a protective role, as the blockade of CP-AMPARs enhanced the seizure-induced potentiation of glutamatergic responses in CA1 [24]. Thus, the exact role of CP-AMPAR incorporation is still unclear.

Multiple reports demonstrate a significant influx of Ca^2+^ during epileptic seizures [25,26,27]. It has been demonstrated that SE elevates the baseline levels of intracellular Ca^2+^ [28]. Studies utilizing in vitro models indicate that epileptiform discharges cause transient increases in intracellular Ca^2+^ concentration [29,30]. Increased intracellular Ca^2+^ concentration can potentially upregulate several Ca^2+^-dependent ion channels, constituting the neuronal membrane’s input conductance. CP-AMPARs that emerge following a period of seizures provide a significant source of Ca^2+^ entry to the neurons [16]. 

We hypothesize that the inclusion of CP-AMPARs should not only enhance excitatory synaptic transmission in the slice but also alter intrinsic membrane properties through calcium-dependent mechanisms, thus modifying the pattern of the ongoing epileptiform activity. However, to our knowledge, no reports concerning the mechanism of the effect of CP-AMPAR on epileptiform activity have been published. The entorhinal cortex plays an essential role in seizure generation in temporal lobe epilepsy [31,32]. In our previous study, which utilized the same preparation, we demonstrated that CP-AMPARs emerge in synapses of the principal neurons of the entorhinal cortex after several minutes of epileptiform activity [18]. Herein, we investigate the immediate action of CP-AMPAR blockade on the generation of epileptiform discharges.

Our results demonstrate that the blocking of CP-AMPARs by N,N,N-trimethyl-5-[(tricyclo [3.3.1.1^3,7^]dec-1-ylmethyl)amino]-1-pentanaminium bromide hydrobromide (IEM-1460) augments the epileptiform activity through a decrease in membrane input conductance. We clarify the contribution of activity-dependent shunting conductance into discharge generation using a mathematical model. The proposed model stems from our previous minimal model of repeating seizure-like events (SLE) and short discharges, called Epileptor-2 [33]. Here we focus not on the SLEs but on the recurrent discharges, which resemble the activity observed during the SE. The model illustrates how the activity-dependent changes of membrane conductance shape the time-course of epileptic discharges.

## 2. Results

### 2.1. Epileptiform Activity Increases the Membrane Input Conductance of the Entorhinal Neurons

**Figure 1:**
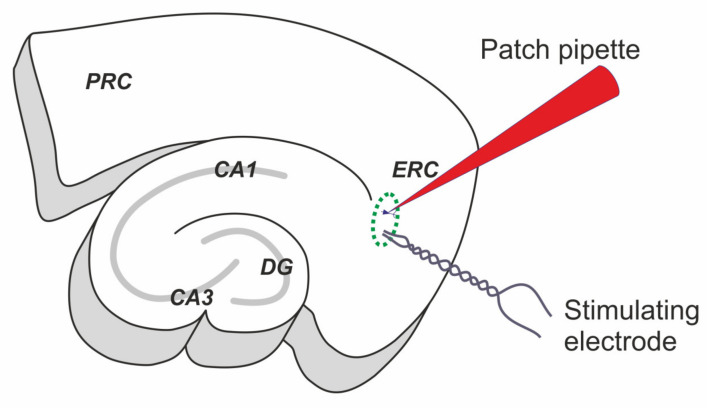
The experimental configuration. The scheme represents the horizontal 350-µM-thick rat brain slice and the location of stimulating and recording electrodes. The whole-cell patch-clamp recordings were performed in the deep layers of the medial entorhinal cortex (ERC), marked with a green oval. PRC—perirhinal cortex, DG—dentate gyrus. CA1 and CA3 are the regions of the hippocampus.

**Figure 2:**
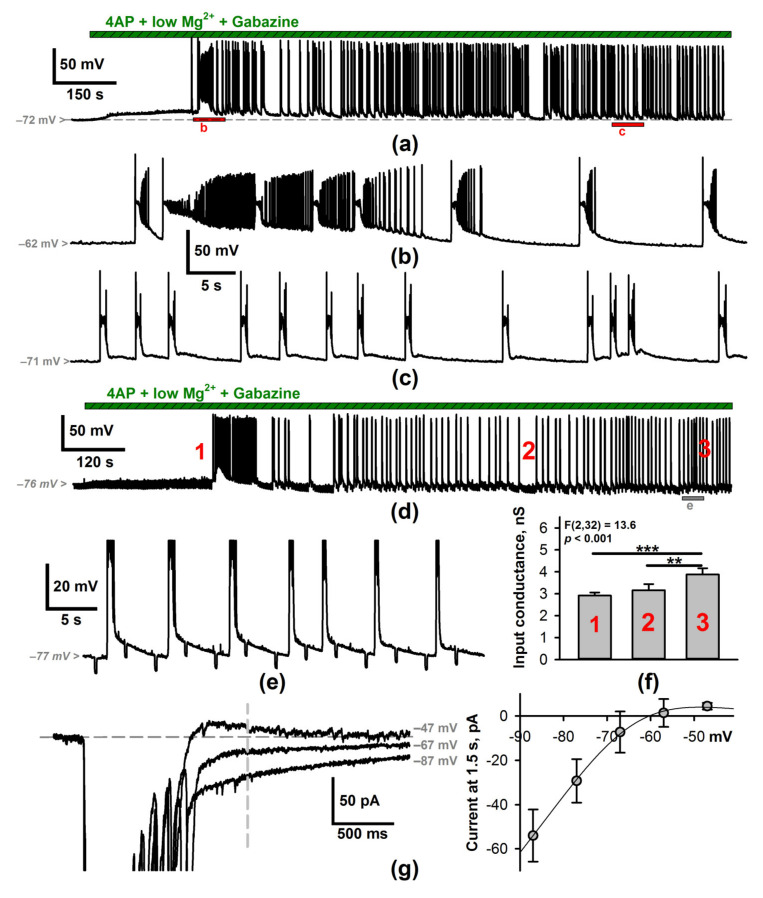
The properties of epileptiform activity. (**a**) A representative current-clamp recording of an entorhinal cortex neuron during the perfusion of the slice with the proepileptic solution. Red bars indicate the fragments, extended at (**b**,**c**). (**b**) Seizure-like event (SLE) and (**c**) steady-state discharges. (**d**) Estimations of the input conductance during the current-clamp recording of an entorhinal cortex neuron. Hyperpolarizing current steps (–25 pA, 250 ms) were applied every 3 s to estimate the input conductance. The red digits indicate the time intervals when the input conductance is calculated. The gray bar indicates the fragment, extended at (**e**). (**f**) The diagram represents the input conductance estimations, which correspond to the time intervals, marked with the red numbers at (**d**). The input conductance increased during the discharge generation. During the prolonged spontaneous pause, its value returned to the initial value (one-way repeated measures ANOVA, *p* < 0.001, followed by the Tukey’s post hoc test (** *p* < 0.01, *** *p* < 0.001)). (**g**) The left panel shows the superimposed voltage-clamp recordings (K-gluconate-based pipette solution) of currents that emerge after the discharge. The vertical dashed line indicates the moment in time when the current value for the I-V curve on the right panel was obtained. The right panel shows the averaged I-V relationship for the current, which emerges after the discharge (*n* = 6). Note that the current reverses at –60 mV and has a pronounced inward rectification. The data were fitted with the following equation: $I\left(v\right)=2.25\;f\left(v\right)\left(v+60.28\right)$, where the function $f\left(v\right)$ determines the voltage-dependence of the membrane conductance and is described by the Boltzmann equation: $f\left(v\right)={\left(1+\exp\left[\frac{\left(v+65.63\right)}{9.82}\right]\right)}^{-1}$.

We performed the whole-cell patch-clamp recordings of the principal neurons of the deep entorhinal cortex in rat brain slices. Slices were perfused with the proepileptic solution with a decreased concentration of Mg^2+^, 4-aminopyridine, and gabazine (Figure 1). The epileptiform activity emerged after several minutes of perfusion (Figure 2a). In most cases, it started with a single SLE (Figure 2b), which transitioned to the generation of short discharges. Within 10–15 min after the first SLE, the duration and frequency of the discharges stabilized (frequency 0.21 ± 0.03 Hz, duration 0.48 ± 0.13 s, *n* = 13, Figure 2c). Since the generation of such discharges lasted about 2 h, we referred to them as steady-state discharges (SSDs). Such a continuous generation of short discharges can be considered an SE model [7,34].

Neurons become hyperpolarized during the generation of SSDs. Before the SLE, the membrane potential was −68.7 ± 1.2 mV, while in the SE regime before each SSD, it became −74.7 ± 1.5 mV (*n* = 10; *p* < 0.01, paired *t*-test). We hypothesized that the activation of an additional potassium conductance mediates this hyperpolarization and would manifest itself in increasing neuronal input conductance.

To test whether the input conductance (Equation (1)) changes during epileptiform activity, we measured it at three stages (Figure 2d): (1) before the first epileptiform discharges; (2) during the prolonged (>10 s) spontaneous pause between the SSDs; (3) during the regular generation of SSDs. Indeed, the input conductance was larger during the SSDs than before epileptiform activity or within the spontaneous pause (Figure 2f, one-way repeated measures ANOVA, F(2,32) = 13.6, *p* < 0.001).

Each SSD is followed by a slow afterdepolarization (sADP, Figure 2e). We used the voltage-clamp recordings of SSDs to plot the current-voltage relationship (I-V relationship) for sADP with the current measured 1.5 s after each SSD. The baseline current before the discharge initiation was subtracted from the current obtained after the discharge, which allowed for the characterization of the transient inactivating component of the activity-induced conductance.

The obtained I-V relationships (Figure 2e) reversed at −63 ± 5 mV (*n* = 6) and displayed an inward rectification (the rectification index was 0.29 ± 0.09, *n* = 6). The obtained reversal potential value is more depolarized than the overall hyperpolarization described above (−74.7 mV), which indicates that sADP is mediated by different ion channels with faster kinetics and weaker ionic selectivity.

**Figure 3:**
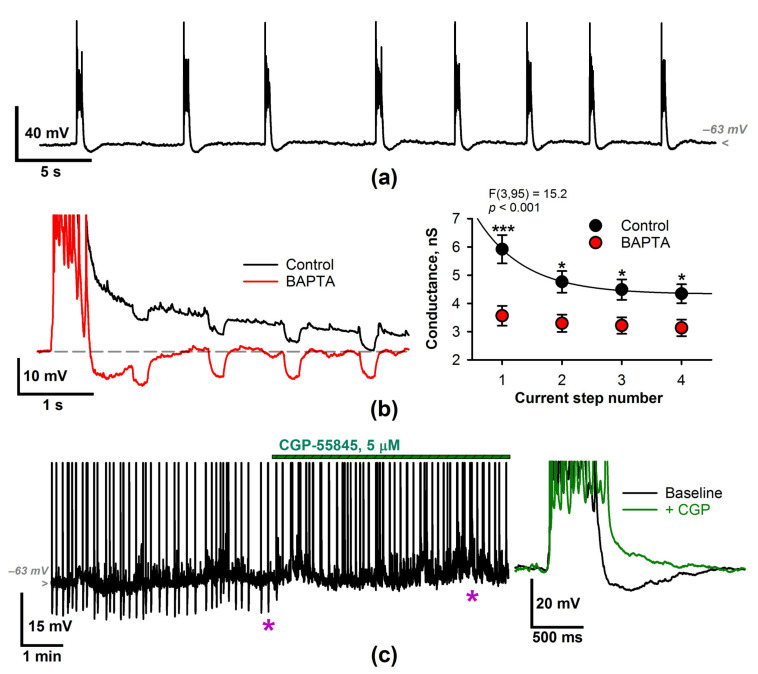
Intracellular calcium buffer BAPTA increases the membrane input conductance. (**a**) A representative current-clamp recording of SSDs obtained with an internal solution containing 10 mM BAPTA. Note the lack of slow ADPs after the discharges. (**b**) The analysis of membrane input conductance after the discharge. The left panel shows two discharges obtained with and without intracellular BAPTA. Discharges were adjusted to a common baseline. Hyperpolarizing current steps (−25 pA, 250 ms) were applied every second following the termination of the discharges. The right panel shows the input conductance after the discharge termination. A single exponential function was fitted to the control data: $f\left(t\right)=A_{\mathrm{baseline}}+A_{\mathrm{transient}}\exp\left[-t/\tau_{\mathrm{transient}}\right]$, where $\tau_{\mathrm{transient}}$ = 920 ± 70 ms, $A_{\mathrm{baseline}}$ = 4.1 ± 0.4 nS and $A_{\mathrm{transient}}$ = 1.5 ± 0.3 nS (*n* = 13). Intracellular BAPTA significantly decreases the input conductance (mixed-design ANOVA, *p* < 0.001, followed by Tukey’s test, * *p* < 0.05; *** *p* < 0.001, *n* = 15 and 9 for control and BAPTA, respectively). (**c**) The transient afterhyperpolarization observed after the discharges recorded with the BAPTA-containing solution was blocked by the GABAbR antagonist CGP-55845. The right panel contains two representative discharges marked with purple asterisks.

As known, Ca^2+^ entry during epileptiform discharges activates the slow K^+^ conductances in hippocampal neurons [35,36]. Therefore, we loaded neurons with the fast calcium buffer 1,2-bis(o-Aminophenoxy)ethane-N,N,Nʹ,Nʹ-tetraacetic Acid (BAPTA) and tested their membrane voltage and input conductance (Figure 3). In the BAPTA-loaded cells, the average membrane potential before the discharge was higher than in cells with a regular intracellular solution (BAPTA: −66.9 ± 1.3 mV, *n* = 17; control: −74.7 ± 1.5 mV, *n* = 10; *p* < 0.001, *t*-test). Furthermore, in most BAPTA-loaded cells, we observed no ADPs after the discharges (Figure 3a).

We injected a series of hyperpolarizing current steps at 1 s intervals and plotted the membrane input conductance versus the time since SSD (Figure 3b). In control, the input conductance exponentially decreased with time. However, in the presence of BAPTA, input conductance was stable and significantly lower (mixed-model ANOVA, F(3,95) = 15.2, *p* < 0.001). Thus, membrane conductance depends on the activation of calcium-dependent ion channels.

Half of the BAPTA-loaded neurons exhibited afterhyperpolarization (AHP) following SSDs (Figure 3c). This AHP disappeared in the presence of GABAbR antagonist CGP-55845 (5 µM), suggesting that it is mediated by the GABAbR-activated inwardly rectifying K^+^ (Kir) channels.

### 2.2. The Effect of a CP-AMPAR Blockade on the Generation of SSDs

**Figure 4:**
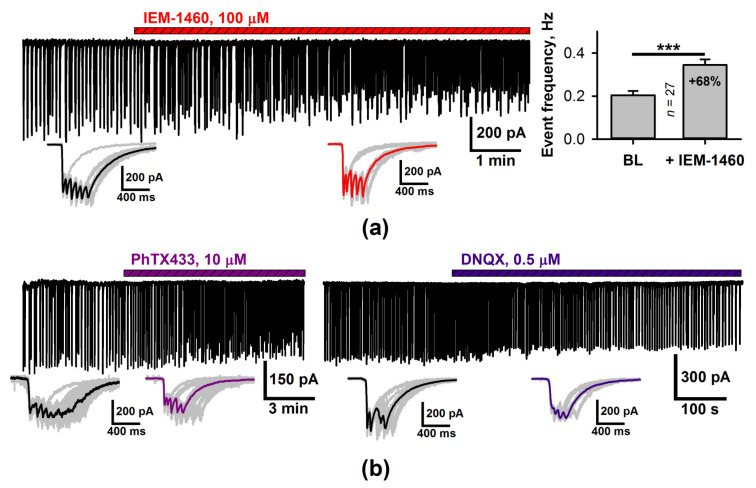
The effect of CP-AMPAR blocker IEM-1460 on SSDs. (**a**) The voltage-clamp recording (V_hold_ = −27 mV) of the entorhinal neuron during the generation of the SSDs. The application of CP-AMPAR antagonist IEM-1460 increases discharge frequency (*** *p* < 0.001, *t*-test). The insets below the trace contain the superposition of the synaptic currents during the individual SSDs. Black and red lines indicate the average discharges before and after the application of IEM-1460. (**b**) Ph-TX433 increases event frequency (left panel) while the application of DNQX at low concentration decreases it (right panel).

Next, we investigated the effect of CP-AMPAR blockade on the generation of SSD by performing the voltage-clamp recordings of the synaptic currents, which are activated during the discharges. Bath application of the selective antagonist IEM-1460 resulted in a 68% increase in SSD frequency (Figure 4a). A comparable effect was observed after the application of philanthotoxin 433 (PhTX433), another antagonist of CP-AMPARs (Figure 4b). The effect of the increase of activity caused by the reduction of excitation is non-trivial. We further elaborate on whether the effect is specific to calcium permeability or not.

The application of IEM-1460 also induced a significant decrease of the maximal amplitude of the synaptic currents activated during the SSDs (at −27 mV the maximal amplitude was −276 ± 25 pA in control and −224 ± 23 pA in the presence of IEM-1460, *n* = 13; *p <* 0.01, paired *t*-test). As CP-AMPARs constitute only a fraction of the total pool of AMPARs, we blocked a similar fraction of AMPARs with 6,7-Dinitroquinoxaline-2,3(1H,4H)-dione (DNQX, 0.5 µM). In contrast to IEM-1460, the application of DNQX at low concentration leads to a decrease in SSD frequency (Figure 4b, right panel; the average discharge frequency was 0.22 ± 0.03 Hz in control and 0.12 ± 0.02 Hz in the presence of DNQX, *n* = 5; *p* = 0.007, paired *t*-test). Thus, the SSD frequency increase after the application of IEM-1460 is specific to CP-AMPARs but not all AMPARs. This suggests that the SSD generation is calcium-dependent because of the CP-AMPARs.

**Figure 5:**
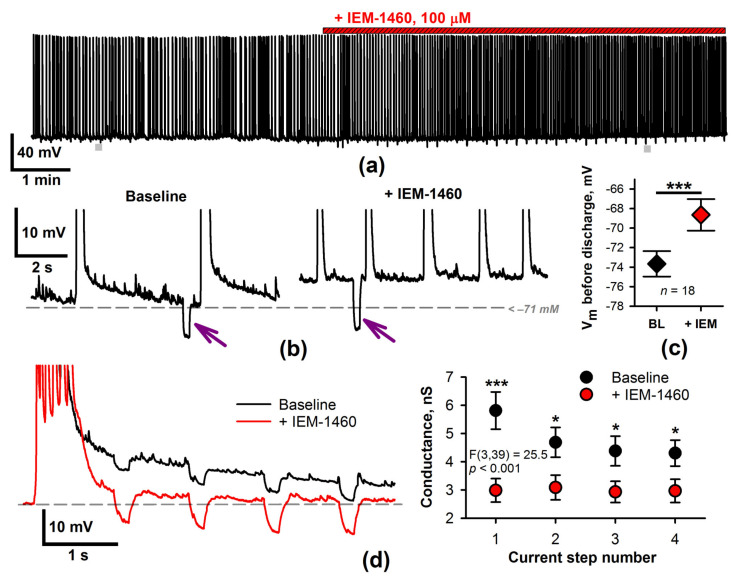
CP-AMPAR blocker IEM-1460 decreases the membrane input conductance and increases the membrane voltage. (**a**) A representative current-clamp recording demonstrates the effect of the IEM-1460 application on epileptiform activity. To estimate input conductance, hyperpolarizing current steps were applied. Gray bars indicate two fragments extended at (**b**). Arrows indicate voltage response to a current step. (**c**) IEM-1460 increases the membrane potential before the epileptiform discharges (paired *t*-test, *p* < 0.001, *n* = 18). (**d**) Analysis of membrane input conductance after discharge. The left panel shows two representative discharges before and after the IEM-1460 application. Hyperpolarizing current steps were applied every second following the termination of the discharges. The right panel shows the input conductance after the discharge termination before and after IEM-1460 administration. IEM-1460 significantly decreases the input conductance value (two-way repeated-measures ANOVA, *p* < 0.001, followed by Tukey’s post hoc test (baseline vs. IEM-1460), * *p* < 0.05, *** *p* < 0.001).

CP-AMPARs provide a significant source of intracellular calcium ions during epileptiform activity [16]. As neuronal membrane potential and input conductance were altered in the presence of BAPTA, we investigated whether these characteristics are affected by the blockade of CP-AMPARs (Figure 5). Indeed, the application of IEM-1460 exerted similar effects as intracellular BAPTA. The average membrane potential before SSD increased (Figure 5b,c), and, in most cases, the ADPs were reduced or abated (Figure 5b). Contrary to the experiments with intracellular BAPTA, no GABAbR-mediated afterhyperpolarization was obtained after SSDs, indicating a decrease of GABAergic interneuron activity in the presence of IEM-1460.

IEM-1460 reduced the input conductance (two-way repeated-measures ANOVA, F(3,39) = 25.5, *p* < 0.001, Figure 5d). These results suggest a decrease in calcium-dependent conductance after administration of the CP-AMPAR blocker.

**Figure 6:**
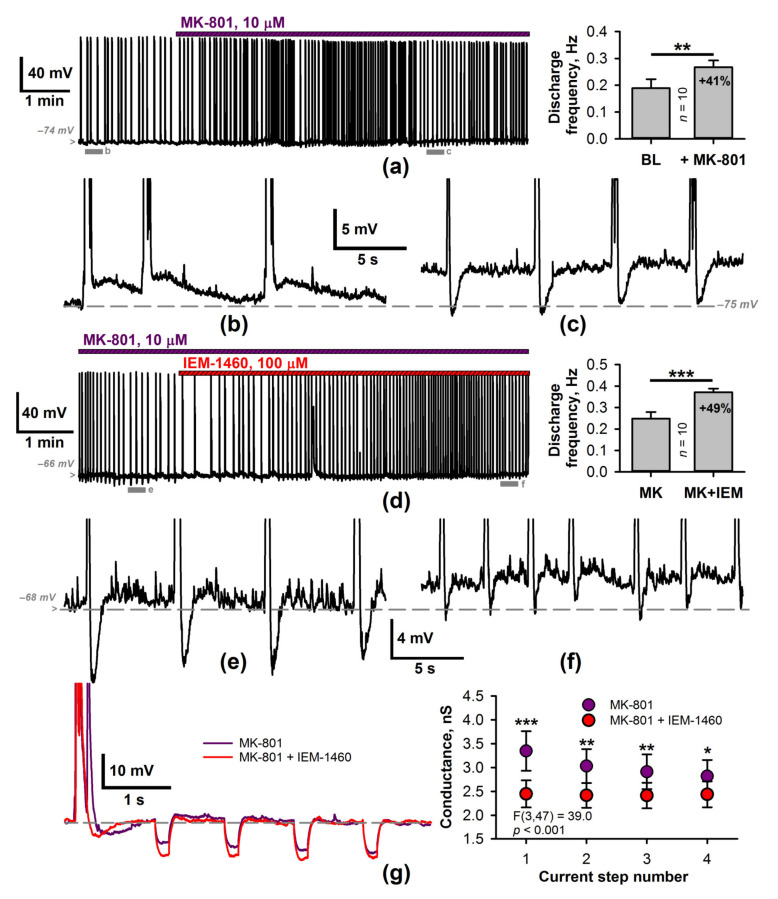
The effects of NMDAR blockade on epileptiform activity. (**a**) Left: a representative current-clamp recording illustrating the effect of MK-801 on epileptiform activity. Gray bars mark the representative regions, extended on (**b**) and (**c**). Right: the average discharge frequency significantly increased following the application of MK-801 (paired *t*-test, *p* < 0.01). MK-801 increased membrane potential and blocked ADPs. (**d**) Representative current-clamp recording illustrates the effect of IEM-1460 in the presence of MK-801. Gray bars indicate the fragments before and after IEM-1460 application and are extended at (**e**) and (**f**), respectively. Right: the average discharge frequency significantly increased following IEM-1460 application (paired *t*-test, *** *p* < 0.001). (**g**) The membrane input conductance after discharge. Discharges recorded in the presence of MK-801 before and after the IEM-1460 application. Hyperpolarizing current steps were applied every second. Right panel: diagram showing changes in input conductance. IEM-1460 significantly decreases the input conductance (two-way repeated-measures ANOVA followed by Tukey’s post hoc tests, * *p* < 0.05, ** *p* <0.01, *** *p* < 0.001).

In addition to CP-AMPARs, there are other potential sources of Ca^2+^ entry during the epileptiform bursts: NMDARs and voltage-dependent Ca^2+^ channels. It was previously reported that afterpotentials following epileptiform events in the entorhinal cortex depend on NMDAR activation [37]. Also, NMDAR-mediated elevation of Ca^2+^ concentration increases the potassium ion conductance in neurons [38]. Hence, next, we confirmed the lack of nonselective inhibition of NMDARs by IEM-1460 (Appendix A) and investigated the effects of the blockade of the NMDARs on epileptiform activity (Figure 6). Bath application of MK-801 (10 µM) significantly increased SSD frequency (Figure 6a) and reduced the input conductance after SSD considerably compared to the control (the datasets from Figure 3b (black dots) and Figure 6g (purple dots) were compared using a mixed-design ANOVA, F(3,107) = 25.2, *p* < 0.001; followed by Dunnett’s post hoc test). In most cases (90%), the slow ADPs that followed the SSDs disappeared, and transient AHPs emerged after MK-801 application (Figure 6b,c). The AHP was comparable to that observed in the presence of intracellular BAPTA (Figure 3) and was blocked by the antagonists of GABAbRs.

Next, we investigated whether the effect of IEM-1460 on the discharge frequency and the membrane input conductance was occluded by the preliminary blockade of NMDARs (Figure 6d–g). In the presence of MK-801, the frequency of the SSDs increased following the blockade of CP-AMPARs (Figure 6a). In addition, IEM-1460 either decreased or eliminated the GABAbR-mediated AHP (Figure 6b,c). In addition, the values of the input conductance were also significantly reduced (Figure 6g). These results indicate that despite the blockade of NMDAR-mediated Ca^2+^ entry into the cell, a fraction of activity-dependent conductance was still present and could be blocked by the CP-AMPAR antagonist.

**Figure 7:**
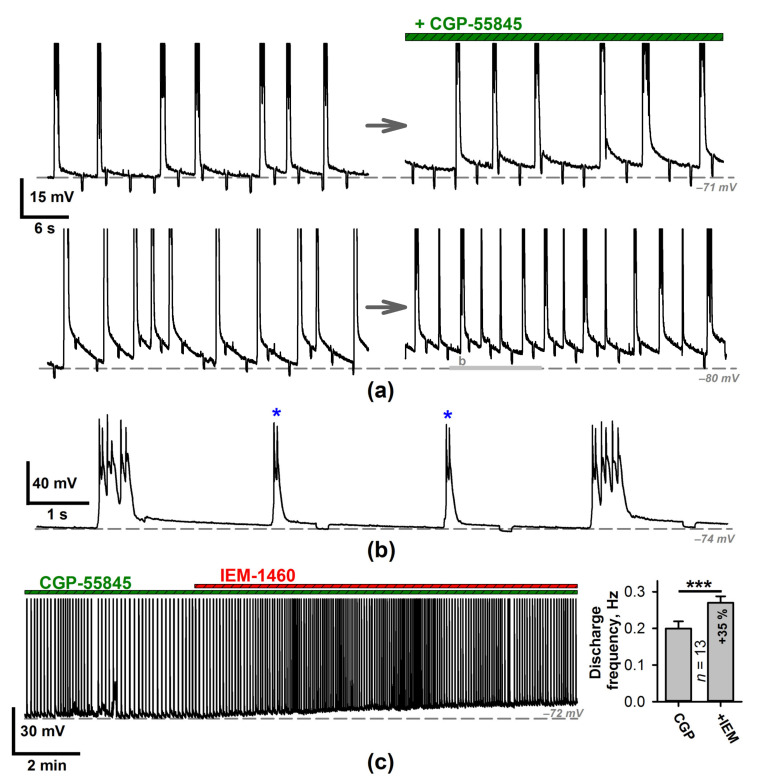
The effects of the GABAbR blockade on epileptiform activity. (**a**) The two representative current-clamp recordings of epileptiform activity before (left) and after (right) application of CGP-55845. The lower example illustrates the case when the abnormally short discharges emerge in between the SSDs. The gray bar indicates the fragment extended at (**b**). Blue asterisks mark the short discharges, which are not present before the application of the GABAbR antagonist. (**c**) Left: the representative current-clamp recording of the epileptiform activity. The preliminary blockade of GABAbRs did not occlude the effect of IEM-1460 application. Right: the average frequency of the discharges increased following IEM-1460 application (*** *p* < 0.001, paired *t*-test,).

CP-AMPARs are normally expressed in GABAergic cortical interneurons [5,6,39,40]. Thus, IEM-1460 can reduce the interneuronal firing and the consequent release of GABA. Due to the presence of gabazine in the perfusing solution, GABAaRs do not affect neurons. However, IEM-1460 blocks GABAb-mediated responses in the presence of intracellular BAPTA or MK-801. Thus, next, we investigated whether the reduced GABAbR activation contributed to the IEM-1460-mediated frequency increase (Figure 7).

Bath application of GABAbR antagonist CGP-55845 did not affect the discharge frequency (the average frequencies before and after CGP-55845 application were 0.18 ± 0.03 Hz and 0.20 ± 0.02 Hz, respectively, *n* = 9, paired *t*-test, *p* = 0.64). However, in 6 of 9 slices, the abnormally short (100–200 ms) discharges emerged between the stereotypical discharges (Figure 7a,b). The application of IEM-1460 after the preliminary blockade of GABAbRs resulted in a significant increase in the discharge frequency (Figure 7c, paired *t*-test). Thus, the prior application of GABAbR antagonists did not occlude the effect of IEM-1460. These results indicate that the proepileptic effect of IEM-1460 cannot be due to a decrease in GABAbR activation.

### 2.3. The Input Conductance Decrease following IEM-1460 Application Results in an Increased Probability of Discharges

**Figure 8:**
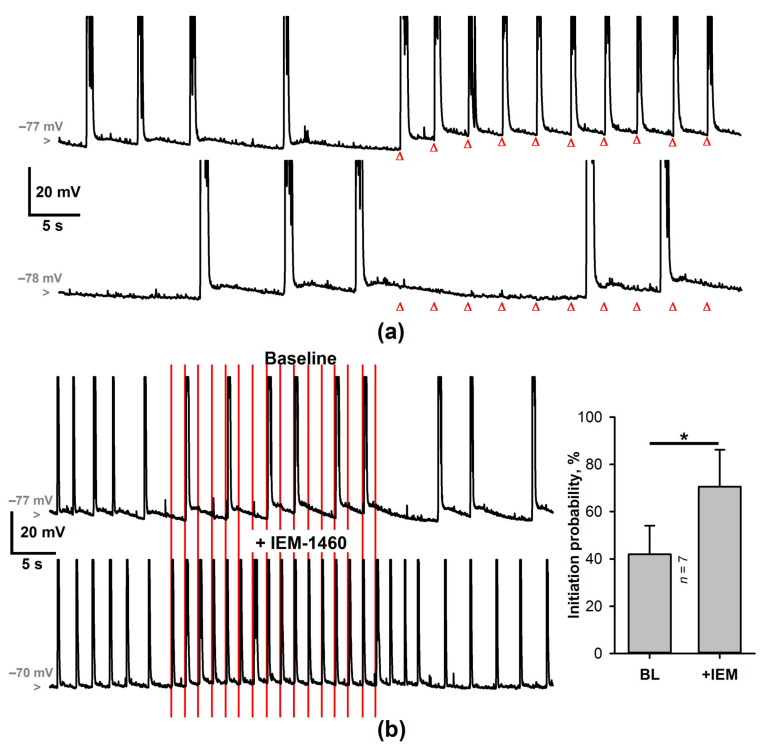
IEM-1460 increases the probability of discharge initiation. (**a**) Two current-clamp recordings from the same representative experiment. Upper trace: extracellular stimulation at 0.5 Hz using 200-µA current pulses initiates the SSD with a 100% probability. Lower trace: the same stimulation with 50-µA current pulses fails to induce any epileptiform discharges. (**b**) Left: two current-clamp recordings from the same representative experiment. In control conditions (upper trace), extracellular stimulation with 100-µA current pulses induces epileptiform events after only 6 of 16 stimuli. After IEM-1460 application (lower trace), the same stimulation induces epileptiform events with 100% probability. Right: the average event initiation probability increases following the IEM-1460 application (* *p* < 0.05, paired *t*-test).

To investigate the effects of additional conductance on discharge generation, we evoked SSDs by the extracellular stimulation (Figure 8). The stimulation frequency was set at 0.5 Hz, which exceeded the frequency of SSDs in the presence of IEM-1460. The probability of discharge initiation depended on the applied current (Figure 8a). We selected the stimulation intensity that would cause the SSDs to occur with 40–50% probability (Figure 8b, upper trace). SSDs were nearly twice as likely after the application of IEM-1460 (Figure 8b,c, paired *t*-test, *p* < 0.05). Thus, the additional Ca^2+^-dependent conductance shunts the response, which could otherwise initialize an SSD.

### 2.4. Simulations of the Epileptiform Activity

Our experiments point to a set of factors that determine the probability of SSD generation and suggest that the Ca^2+^-dependent shunt explains the effect of CP-AMPARs on the discharge frequency. We address whether this representation is feasible with the modeling study. We elaborated a mathematical model of epileptiform activity (Equations (2)–(22)), which further developed our previous model, “Epileptor-2” [33].

Several modifications were introduced. First, inspired by the noise observed in Figure 5b in the intervals between the discharges, we simulated more realistic, non-Gaussian noise by introducing a specific neuronal population that generates spontaneous activity. Thus, we distinguished two neuronal populations: one represents most glutamatergic neurons, and the other is a localized group of neurons generating random synchronized bursts. These bursts may trigger an SSD in the first population. The probability of recruiting neurons in the first population depends on the fraction of available NMDAR and AMPAR conductances and the neuronal input conductance.

The second essential modification is the addition of firing rate-dependent conductance that reflects activation of the calcium-dependent channels (Equations (16) and (17)) found in the present study (Figure 3b). The additional conductance includes two components: persistent and transient. The overall hyperpolarization during the regular discharge generation indicates that K^+^ channels mostly mediate the persistent conductance. Its decay time constant was set to $\tau_{pers}\;$= 35 s. The transient component decay time was $\tau_{tr}$ = 0.92 s as it was established in Section 2.1. The voltage-clamp experiments (Figure 2g) demonstrated that the transient current was voltage-dependent with the corresponding reversal potential equal to a linear combination of $V_{Na}$ and $V_{K}$.

The third essential modification concerns the mechanism of discharge termination. In the original “Epileptor-2,” the variable “synaptic resource” was responsible for the termination of epileptiform discharge: it decreases during neuronal firing and is gradually restored between the bursts. As the excitatory input became overpowered by the inhibitory input, the discharge terminated. In the current model, the inhibitory synaptic input is lacking, as the experimental recordings were performed in the presence of gabazine, the antagonist of GABAaRs. The “synaptic resource” was subdivided into two components with different activation and inactivation kinetics — $\chi^{syn}$ and $\chi^{NMDA}$. These variables are governed by the same equations from the original model and represent the overall short-term use-dependent synaptic exhaustion and the fraction of available NMDARs, respectively. The kinetics of the $\chi^{NMDA}$ was modeled to be significantly slower than that of $\chi^{syn}$. As a result, the new model incorporates three mechanisms underlying the termination of each of the discharges: (1) the use-dependent exhaustion of excitatory synapses; (2) the shunting effect of the input conductance, which increases during the discharge; and (3) the impact of increased current through the Na^+^/K^+^ pump due to the accumulation of ${\left[K^{+}\right]}_{o}$ and ${\left[Na^{+}\right]}_{i}$ during the discharges. Only mechanisms 1 and 3 were implemented in the original “Epileptor-2,” and mechanism 3 was effective for SLEs only.

**Figure 9:**
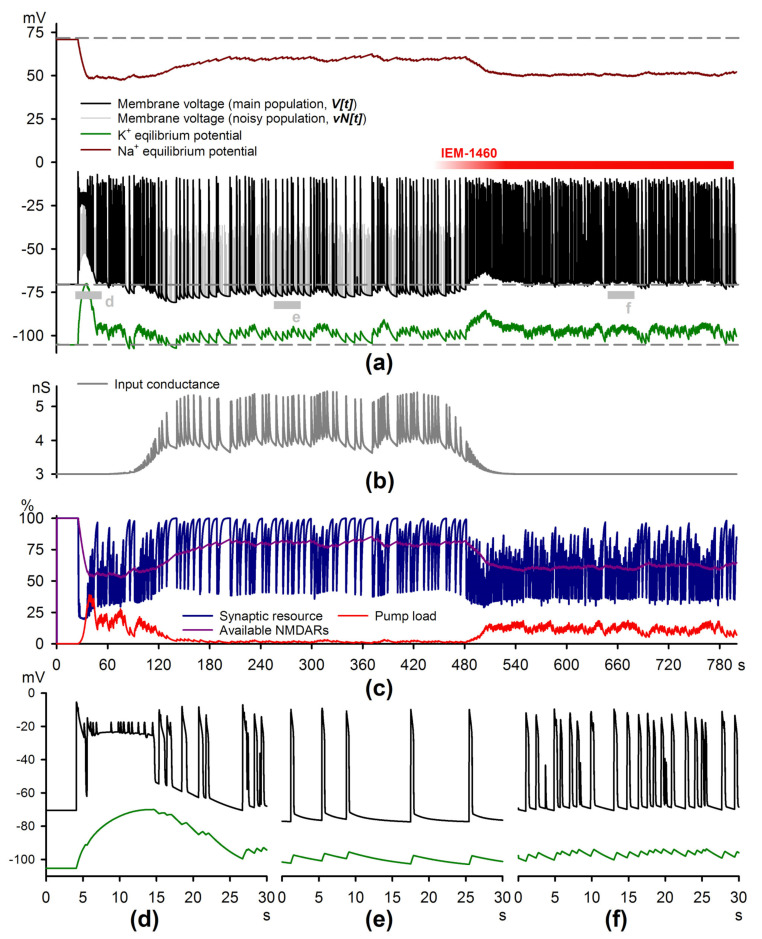
Simulation of the epileptiform activity. (**a**) The plots of the membrane voltages of two neuronal populations (black and gray lines for variables V and $V_{Noisy}$, respectively), and the reversal potentials for the potassium and sodium ions (dark red and green lines for $V_{Na}$ and $V_{K}$, respectively). Suprathreshold depolarizations represent epileptiform discharges. Note the significant increase of the discharge frequency following the application of IEM-1460 and the overall hyperpolarization observed after the first initial SLE until the application of the drug. Gray bars indicate the fragments, extended at (**d**–**f**); (**b**) The total membrane conductance, calculated as the sum of the leak conductances and activity-dependent conductances (Equation (22)). The latter is allowed to be activated during the time window beginning after the initial SLE and ending after the application of IEM-1460. (**c**) The plots representing the dynamics of the variables $\chi^{syn}$, $\chi^{NMDA}$, and Na^+^/K^+^ pump current (as a fraction of the maximal achievable current $I_{pump,MAX}$). The fast dynamics of $\chi^{syn}$ contributes to the termination of the discharge, while the slow dynamics of the $\chi^{NMDA}$ determines the fraction of available NMDARs. Note that the latter decreases following the application of IEM-1460. (**d**–**f**) represent the extended fragments from (**a**), corresponding to the initial SLE (**d**), SSD (**e**), and SSDs after IEM-1460 application (**f**).

The modified model reproduces the evolution of epileptiform activity (Figure 9a,d,e to be compared with Figure 2a–c) and the effect of IEM-1460 application (Figure 9a,f and Figure 5a). In an experiment, one-two SLEs were observed at the beginning. As the ${\left[Na^{+}\right]}_{i}$ increases at a slower rate than ${\left[K^{+}\right]}_{o}$ due to the difference of intracellular and extracellular volumes, the activity of the Na^+^/K^+^ pump rises with a slight delay after the K^+^ transient (Figure 9c red trace vs. Figure 9a green trace), which allowed for the level of ${\left[K^{+}\right]}_{o}$ to stay elevated for tens of seconds, leading to the SLE. As the Na^+^/K^+^ pump eliminates the initial elevation of ${\left[K^{+}\right]}_{o}$, the SLE slowly transitions to the regular generation of late-stage discharges. At this stage, the activity-dependent conductances emerge (Figure 9b). As these conductances fully develop, the generation of the short discharges maintains, which is comparable to the regime of experimentally observed SSDs (Figure 2c). Each of the SSDs was followed by the slow ADP, resulting from both the transient increase of ${\left[K^{+}\right]}_{o}$ during the discharge and the activation of transient conductance, the current through which has the reversal potential of about −60 mV.

The application of IEM-1460 was modeled as a 30% decrease of AMPAR conductance and a 100% blockade of activity-dependent conductances. Both blocks were induced using smooth sigmoid-shaped functions (Equations (11) and (18), half-maximal block was introduced at 450 s for AMPARs and 480 s for additional conductances). As a result, the pattern of discharge generation changed in a manner comparable to experimental observations (Figure 4a and Figure 5a): a significant increase of the discharge frequency accompanied by the overall depolarization and the reduction of ADPs was observed. The latter is a consequence of both altered ${\left[K^{+}\right]}_{o}$ dynamics (the amplitude of the K^+^ transients decreased, while the baseline level increased), and the blockade of the activity-dependent conductances. An increased frequency of epileptiform bursts reduces the fraction of available NMDARs (Figure 9c, purple line).

The obtained results highlight the new protective role for the inclusion of CP-AMPARs during seizures: these receptors help to sustain the activity-dependent conductances at a high level, which reduces the severity of the SE in the implemented model.

## 3. Discussion

**Figure 10:**
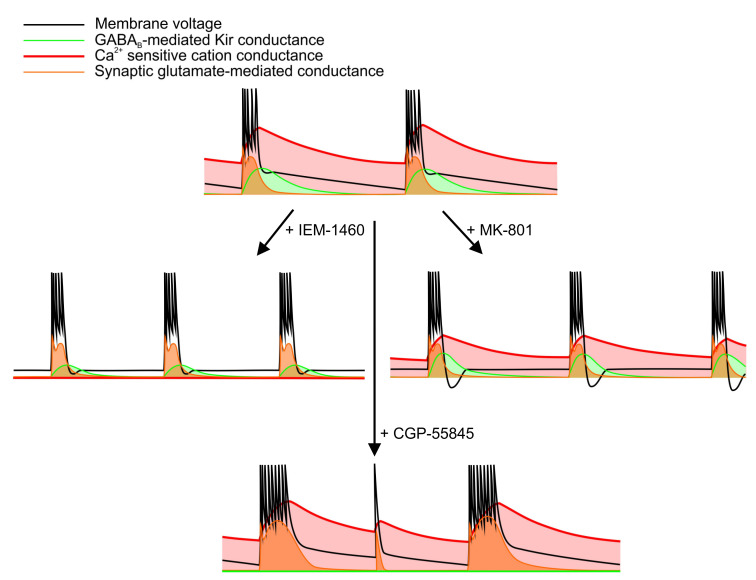
The scheme summarizes the effects of the drugs utilized in the experiments. In the control conditions, three major types of conductances affect the generation of epileptiform bursts in the used in vitro model: the synaptic conductance mediated by the AMPARs and NMDARs (orange line), the Ca^2+^-dependent conductance, which is activated during the discharge and gradually decreases between them (red line), and the GABAb-mediated Kir conductance that is present for a brief period after the discharge (green line). After the bath application of IEM-1460, these conductances are significantly reduced. As a result, shorter bursts that are not followed by the ADPs are generated at a higher frequency. The use of MK-801 leads to a significant decrease in synaptic conductance but affects Ca^2+^-sensitive and GABAb-mediated conductances to a lesser extent than the use of IEM-1460. As a result, the Kir-mediated AHPs manifest themselves in the recordings of the membrane voltage. Finally, the application of CGP-55845 blocks potassium conductance, which contributes to the discharge termination. As a result, the duration of the discharges increases, and undeveloped discharges occur between them.

In the current study, we analyzed the effects of CP-AMPAR blockade on the SE-like activity in an in vitro model. IEM-1460 increases the discharge frequency by decreasing the input conductance of neurons, which is maintained at a high level due to Ca^2+^ entry during the epileptiform discharges. Figure 10 sums up the impact of the drugs used in the current study: IEM-1460, MK-801, and CGP-55845. The obtained results highlight the dual effect of CP-AMPAR incorporation during seizures: on the one hand, these newly expressed receptors contribute to the seizure-induced enhancement of excitatory synaptic transmission. On the other hand, activation of these receptors augments the input conductance of neurons, thus decreasing the overall network excitability. In the utilized preparation, the second effect appears to play a dominant role.

### 3.1. Membrane Conductance as a Factor of Seizure Generation

The membrane input conductance determines the neuron’s excitability [41] and results from the interplay of a large number of ion channel types [42]. However, the majority of studies regarding the mechanisms of epileptiform activity focus either on the properties of synaptic transmission [7,15,43] or on the alterations of the ion dynamics [25,44], but not on the ability of neurons to integrate the incoming synaptic signal. In addition, dynamic changes in input conductance are not accounted for in most existing mathematical models of epileptic seizures. However, several studies [45,46,47], including those conducted in our laboratory [48,49], demonstrate that input resistance can change after seizures.

Epileptic activity can alter neuronal excitability through many potential mechanisms. Ca^2+^ elevations during epileptiform bursts can modulate several types of ion channels. For example, the activation of Ca^2+^-dependent K^+^ channels during the discharges contributes to the slow post-burst afterhyperpolarization, affecting the timing of epileptiform events [35,36,50]. Activation of metabotropic glutamate receptors and Ca^2+^ elevations promote the activity of TRPC and TRPM channels, which underlie the post-burst afterdepolarizations and contribute to the neurons’ intrinsic excitability [51,52,53,54]. Ca^2+^ elevations enhance the activity of HCN channels [55], which can affect the pattern of the epileptiform activity [56] and contribute to the post-burst AHP [57]. The epileptic discharges upregulate ATP-sensitive K^+^ channels, which increased activity may terminate seizures [58,59]. Sodium-activated K^+^ channels upregulation may be the other mechanism of seizure termination [60].

In the current study, we have not identified the exact type of ion channel upregulated due to the Ca^2+^ entry via CP-AMPARs. We hypothesize that at least two types of channels contribute to the epileptiform activity-induced conductance increase: the slow potassium channel, mediating global hyperpolarization, and a faster nonselective channel, contributing to the transient ADPs. The yet unidentified channels which mediate the slow AHP at a time scale of seconds can be responsible for the activity-dependent hyperpolarization [61,62,63]. TRP-mediated afterdepolarizations following a spike train that induce the persistent firing in entorhinal neurons were also described [52,62,64]. The slow AHP and the TPR-dependent ADP interplay might underlie the activity-dependent conductance increase in our preparation. The GABAb-mediated Kir conductance is also present after the discharge termination, though it does not persist long enough to affect the neuronal excitability between the discharges. Thus, the generation of epileptiform discharges is shaped by dynamic changes of neuronal excitability, mediated by several types of slow conductances. Incorporating such changes into existing mathematical models may provide a better understanding of the mechanism of seizures.

### 3.2. The Physiological Role of Abnormal Expression of CP-AMPARs during Seizures

Several reports indicate that CP-AMPARs are expressed in synapses of glutamatergic neurons of the hippocampus and entorhinal cortex during seizures [15,16,18]. Moreover, the brief appearance of CP-AMPARs was reported as a delayed response to epileptic seizures in several experimental models [65,66,67,68,69,70]. Such abnormal expression is usually considered pathological because it can contribute to calcium-dependent cell loss after seizures and the development of acquired epilepsy. Some studies even propose the CP-AMPARs as a therapeutic target [3,23,71,72].

Our previous study demonstrated that CP-AMPARs are not required to form seizure-induced long-term potentiation (LTP) in CA1 [24]. This result indicates that the primary function of CP-AMPAR incorporation during seizures is not the promotion of synaptic plasticity, which is the case for the conventional LTP [73]. The current study demonstrates that the input conductance of neurons displays a substantial sensitivity to Ca^2+^ entry through the CP-AMPARs. Thus, incorporating CP-AMPARs might represent an immediate compensatory mechanism, which decreases neuronal excitability during seizures.

The obtained results contradict several previous reports regarding the effect of IEM-1460 in in vivo models. Intraperitoneal injection of IEM-1460 rapidly terminated SE in mice in a dose-dependent manner [23]. IEM-1460 exerted an antiepileptic effect in immature rats in the pentylentetrazole (PTZ) model and the model of cortical after discharges [21]. However, in the latter model, IEM-1460 had a moderate pro-convulsant effect in 18–25-day-old animals whose age corresponds to the age of the animals used in the present study (21 d). No effect of this drug in the maximal electroshock model was detected [74]. This contradiction between the results obtained in in vitro and in vivo models indicates that in animals, the seizure-induced enhancement of excitatory synaptic transmission might be more pronounced than in brain slices, overpowering the effect of the input conductance increase. Nonetheless, the possibility of using the CP-AMPAR antagonists for the treatment of epileptic seizures requires further investigation.

## 4. Materials and Methods

### 4.1. Animals

Three-week-old male Wistar rats (*n* = 60) were used in this study. The animals were kept under standard conditions with free access to food and water. All animal procedures followed the European Community Council Directive 86/609/EEC and were approved by the Sechenov Institute of Evolutionary Physiology and Biochemistry Bioethics Committee.

### 4.2. Brain Slice Preparation

The rats were decapitated, and their brains were removed rapidly. The brain slice preparation method was described previously [75,76]. A vibrating microtome (Microm HM 650 V; Microm, Walldorf, Germany) was used to cut horizontal 350-µm-thick slices that contained the hippocampus and the adjacent cortical regions (including the entorhinal cortex and the perirhinal cortex; Figure 1). Artificial cerebrospinal fluid with the following composition (in mM) was used: 126 NaCl, 24 NaHCO_3_, 2.5 KCl, 2 CaCl_2_, 1.25 NaH_2_PO_4_, 1 MgSO_4_, and 10 dextrose. The artificial cerebrospinal fluid was aerated with a gas mixture of 95% O_2_ and 5% CO_2_. All chemicals used to prepare the solutions were purchased from Sigma-Aldrich (St. Louis, MO, USA) unless stated otherwise. A total of 1–3 slices per rat were used for the experiments.

### 4.3. In Vitro Model of Epileptiform Activity

Epileptiform activity in rat brain slices was induced using a pro-epileptic solution containing the following (in mM): 126 NaCl, 24 NaHCO_3_, 2.5 KCl, 2 CaCl_2_, 1.25 NaH_2_PO_4_, 0.25 MgSO_4_, 10 dextrose, 0.05 4-aminopyridine, 0.01 gabazine.

### 4.4. The Whole-Cell Patch-Clamp Recordings

The recordings were performed at 30 °C. Pyramidal neurons in the deep layers of the dorsal region of the medial entorhinal cortex were visualized using a Zeiss Axioscop 2 microscope (Zeiss, Oberkochen, Germany) equipped with differential interference contrast optics and a video camera (Grasshopper 3 GS3-U3-23S6M-C; FLIR Integrated Imaging Solutions Inc., Wilsonville, OR, USA). Patch electrodes (3–5 MΩ) were pulled from borosilicate glass capillaries (Sutter Instrument, Novato, CA, USA) using a P-1000 pipette puller (Sutter Instrument, Novato, CA, USA). A cesium methanesulfonate-based pipette solution (composition in mM: 127 CsMeSO_3_, 10 NaCl, 5 EGTA, 10 HEPES, 6 QX314, 4 ATP-Mg, and 0.3 GTP; pH adjusted to 7.25 with CsOH) was used for voltage-clamp recordings. A potassium gluconate-based pipette solution was used both for current- and some voltage-clamp recordings (composition in mM: 136 K-Gluconate, 10 NaCl, 5 EGTA, 10 HEPES, 4 ATP-Mg, and 0.3 GTP; pH adjusted to 7.25 with KOH). For the current-clamp recordings with intracellular BAPTA, a solution of the following composition was used: 130 K-Gluconate, 10 NaCl, 10 BAPTA, 10 HEPES, 4 ATP-Mg, and 0.3 GTP; pH adjusted to 7.25 with KOH.

Whole-cell recordings were performed using a Multiclamp 700B (Molecular Devices, Sunnyvale, CA, USA) patch-clamp amplifier and an NI USB-6343 A/D converter (National Instruments, Austin, TX, USA) using WinWCP 5 software (University of Strathclyde, Glasgow, U.K.). The data were filtered at 10 kHz and sampled at 30 kHz. In all cells included in the sample, access resistance was less than 15 MΩ and remained stable (≤20% increase) across the experiment. The liquid junction potential was compensated offline for the voltage-clamp recordings by subtracting 7 mV. Recoding of only one neuron per slice was performed.

The extracellular stimulation was performed using a bipolar twisted nichrome electrode, which was placed in the same layer as the recorded neuron at a distance of 100–200 µm (Figure 1).

The estimations of the input conductance were performed by applying hyperpolarizing current steps (250 ms, −25 pA). In order to estimate average input conductance over a period of time, the hyperpolarizing current steps were applied every 3 s; 12–15 voltage responses to current steps were accumulated, and their amplitude was averaged; the current steps that coincided with the discharge were discarded). In order to characterize the dynamics of the input conductance after the epileptiform discharge, 4 hyperpolarizing current steps were applied every second, starting 0.5–1 s from the discharge termination; for each slice, estimations were performed after 5–10 randomly selected discharges, and the amplitudes of the voltage responses to the corresponding current steps were averaged. The value of the input conductance ($G_{input}$, nS) was calculated using the Ohms law:

(1) $$G_{input}=I_{step}/\Delta V$$

where $I_{step}$ is the current step amplitude (25 pA), and ∆V is the membrane hyperpolarization (in mV).

The rectification index of the I-V relationship of current, obtained after the discharges, was calculated as the ratio of slopes of the positive and negative parts of the curve.

A selective antagonist of CP-AMPAR IEM-1460 (100 µM) was used throughout this study. IEM-1460 was synthesized and characterized by Yury Skorik (Institute of Macromolecular Compounds, Saint Petersburg, Russia).

### 4.5. Statistics

The data analysis was performed using custom software written in Wolfram Mathematica 12 (Wolfram Research, Champaign, IL, USA). Sigmaplot 14 (Systat Software Inc., San Jose, CA, USA) was used for the statistical analysis and graphical representation of the results. Dixon’s *Q*-test (at the 95% confidence level) was used to reject outliners. The Kolmogorov–Smirnov test was employed for the evaluation of the normality of sample data. The equality of variance was assessed using the Levene median test. For data that had a normal distribution and passed an equal variance test, the statistical significance was assessed using a Student’s *t*-test or ANOVA. Mauchly’s test for sphericity was used before the repeated-measures ANOVA. The mixed-design ANOVA analysis of the neuronal input resistance was performed, considering the stimulus number and the presence of the drug under consideration as the within and between-subject factors, respectively. The results were considered significant when *p* < 0.05. Dunnett’s post hoc test was used for multiple comparisons vs. the control group. The Tukey post hoc test was used for multiple pairwise comparisons. The results were expressed as a mean ± standard error of the mean. The characteristics that correspond to individual epileptiform bursts (such as the discharge duration or the input conductance following the discharge) were reported as grand average values: 20–50 discharges were accumulated for each recorded neuron, and the value of the reported parameter was averaged. In this case, “*n*” represents the number of the recorded neurons.

### 4.6. The Mathematical Model of Epileptiform Activity

The proposed model is an extension of our previously proposed minimal model of epileptiform discharges “Epileptor-2” [33]. The novel model consists of 10 ordinary differential equations of the first order (Equations (2)–(5), (12), (13), (16), (17), (19)–(20)) and supplementary algebraic relationships, written below (Equations (6)–(11), (14), (15), (18), (21)). The main variables are marked in bold font.

We hypothesize that an epileptiform burst is triggered by the synchronous firing of a relatively small subset of neurons (“noisy population”), which can recruit all the other neurons in the slice. Therefore, we assume that neurons may be split into 2 populations, where the first one consists of spontaneously firing neurons and triggers another one that contains the majority of neurons. The membrane voltage of the main population of neurons ***V***(t) is modeled by using the Kirchhoff’s current conservation law:

(2) $$-C\frac{d\;V}{dt}=g_{K,leak}\left(V-V_{K}\right)+g_{Na,leak}\left(V-V_{Na}\right)+g_{Cl,leak}\left(V-V_{Cl}\right)+I_{pump}\left(t\right)\;+G_{trig}\left(t\right)\;\left(V-V_{Glut}\right)+G_{reciprocal}\left(t\right)\;\left(V-V_{Glut}\right)+I_{trans}\left(t\right)+I_{pers}\left(t\right)$$

where C represents the membrane capacitance, $g_{K,leak},g_{Na,leak}$ and $g_{Cl,leak}$: the leak conductances for the potassium, sodium, and chloride ions, respectively. $V_{Glut}$: the reversal potential of the glutamate-mediated currents; it was set equal to 0 mV. $V_{K}$, $V_{Na}$ and $V_{Cl}$ represent the reversal potentials for these ions (in mV), according to the Nernst equations:

$V_{K}=26.1\;mV\;\ln\frac{{\left[K^{+}\right]}_{o}\;}{{\left[K^{+}\right]}_{i}}$, $V_{Na}=26.1\;mV\;\ln\frac{{\left[Na^{+}\right]}_{o}}{{\left[Na^{+}\right]}_{i}}$, $V_{Cl}=26.1\;mV\;\ln\frac{{\left[Cl^{-}\right]}_{i}}{{\left[Cl^{-}\right]}_{o}}$

${\left[K^{+}\right]}_{o}$, ${\left[K^{+}\right]}_{i}$, ${\left[Na^{+}\right]}_{o}$, ${\left[Na^{+}\right]}_{i}$, ${\left[Cl^{-}\right]}_{o}$ and ${\left[Cl^{-}\right]}_{i}$: the intracellular and extracellular concentrations of K^+^, Na^+^, and Cl^-^ ions.

The membrane voltage of the triggering population receives noise. The “noisy” population membrane potential $V_{Noisy}\left(t\right)$ is modeled as follows:

(3) $$-C\frac{d\;V_{Noisy}}{dt}=g_{K,leak}\left(V_{Noisy}-V_{K}\right)+g_{Na,leak}\left(V_{Noisy}-V_{Na}\right)+g_{Cl,leak}\left(V_{Noisy}-V_{Cl}\right)\;+I_{pump}\left(t\right)+G_{noise}\left(V_{Noisy}-V_{Glut}\right)+I_{trans}\left(t\right)+I_{pers}\left(t\right)$$

The noise conductance of the second population $G_{noise}\left(t\right)$ was modeled as

(4) $$\frac{d\;G_{noise}\;}{dt}=-\frac{G_{noise}}{\tau_{noise}}+\underset{i}{\sum }\xi_{i}\;\delta \left(t-t_{i}\right)\;$$

where $\tau_{noise}$ = 25 ms, $\xi_{i}\;$ the random numbers with the mean $\langle \xi_{i}\rangle =\mu$ and the variance $\langle \xi_{i}^{2}\rangle =\sigma^{2}$; the time moments $t_{i}$ satisfy the Poisson distribution with the mean rate λ. These parameters were set as follows: μ= 8 nS, σ= 1.5 nS, λ= 1.5 Hz.

The recruitment of the main population into the activity is determined by the signal transduction from the noisy population, which is governed by the dimensionless variable $T_{syn}\left(t\right)$:

(5) $$\frac{d\;T_{syn}\;}{dt}=\nu_{N}\left(V_{Noisy}\right)\left(1-T_{syn}\right)-\frac{T_{syn}}{\tau_{T}}$$

where $\tau_{T}$ = 25 ms.

The input-output function $\nu_{N}\left(V_{Noisy}\right)$ is a unit step:

(6) $$\nu_{N}\left(V_{Noisy}\right)=\left\{\begin{array}{c} 0,V_{Noisy}<-35\;mV\\ 1,\;V_{Noisy}\geq -35\;mV \end{array}\right.$$

If a noise burst induces a subthreshold depolarization of the noisy population, then the value of $T_{syn}$ becomes equal to 1 and activates the triggering glutamatergic input to the main population, determined by the conductance $G_{trig}\left(t\right)$:

(7) $$G_{trig}\left(t\right)=g_{trig}\;T_{syn}\;\chi^{syn}\;f_{Block}\left(t;t_{block}\right)$$

where $g_{trig}$ represents the maximal conductance set equal to 9.5 nS.

The reciprocal input to the main population is mediated by AMPARs and NMDARs and is determined by the equation:

(8) $$G_{reciprocal}\left(t\right)=g_{Glut}\;\left(0.33\;f_{Block}\left(t;t_{block}\right)+0.67\;f_{NMDA}\left(V\right)\;\chi^{NMDA}\right)\;\chi^{syn}\;\nu \left(V\right)$$

where $g_{Glut}$ represents the maximal conductance set equal to 30 nS. $f_{NMDA}\left(V\right)$ describes the dependence on the voltage of the Mg^2+^-block of NMDAR ion channels [77]:

(9) $$f_{NMDA}\left(V\right)=\frac{1}{1+\exp\left[0.06\;\left(-47.77-V\right)\right]}$$

The parameters of this equation corresponding to the utilized proepileptic solution with low Mg^2+^ concentration were taken from [18].

The input-output function of the main population of neurons is described by a sigmoidal function:

(10) $$\nu \left(V\right)=\frac{1}{1+\exp\left[0.286\;\left(-30-V\right)\right]}$$

The function $f_{Block}\left(t;t_{block}\right)$ describes a gradually induced 30% block of AMPAR-mediated conductance at about $t=t_{block}=$ 450,000 ms, assuming that this fraction of AMPARs corresponds to CP-AMPARs:

(11) $$f_{Block}\left(t;t_{block}\right)=1-\frac{0.3}{1+\exp\left(-t+t_{block}/15,000\right)}$$

The fraction of functionally available glutamatergic receptors is determined by the variables $\chi^{syn}\left(t\right)$ and $\chi^{NMDA}\left(t\right)$, which represent the fast use-dependent depression of the synaptic transmission and specifically the slow use-dependent downregulation of NMDARs, respectively. Their dynamics is described by identical equations [33,78]:

(12) $$\frac{d\;\chi^{syn}}{dt}=-r_{rs}\;\nu \left(V\right)\;\chi^{syn}+\frac{1-\chi^{syn}}{\tau_{rs}}$$

(13) $$\frac{d\;\chi^{NMDA}}{dt}=-r_{rN}\;\nu \left(V\right)\;\chi^{NMDA}+\frac{1-\chi^{NMDA}}{\tau_{rN}}$$

The parameters of these equations were chosen to mimic the glutamatergic discharges observed in the experiments: $r_{rs}$ = 0.003 ms^−1^, $\tau_{rs}$ = 1500 ms, $r_{rN}$ = 0.00007 ms^−1^, $\tau_{rN}$ = 52,000 ms. Thus, the fraction of available NMDARs, $\chi^{NMDA}$, has much slower dynamics than $\chi^{syn}$.

As demonstrated in the Results section, the epileptiform activity increases the input conductance of neurons. This additional activity-dependent conductance splits into two parts: the one that inactivates within several seconds between the discharges (the transient conductance) and the one with a much slower inactivation rate (the persistent conductance). The membrane voltages of both populations are affected by the currents through the ion channels, mediating these conductances. Thus Equations (2) and (3) contain $I_{trans}$ and $I_{pers}$.

$I_{trans}$ is modeled by using the following equation:

(14) $$I_{trans}\left(U\right)=g_{trans}\;\left(m_{K}\left(U-V_{K}\right)+m_{Na}\left(U-V_{Na}\right)\right)\;f_{trans}\left(U\right)\;G_{trans}\left(t\right)\;W\left(t\right)$$

where U stands for either $V\left(t\right)$ or $V_{Noisy}\left(t\right)$. As found in the Results section, the transient current has the reversal potential of about −62 mV, thus $m_{K}$ and $m_{Na}$ were set equal to 0.8 and 0.2, respectively. $g_{trans}$ was set equal to 2 nS according to typical experimental estimates; and the voltage-dependence $f_{trans}\left(V\right)\;$ is established in the Results (Section 2.1, Figure 1).

$I_{pers}$ is modeled as a K^+^ current:

(15) $$I_{pers}\left(U\right)=g_{pers}\;G_{pers}\left(t\right)\;\left(U-V_{K}\right)\;W\left(t\right)$$

where $g_{pers}$ was set equal to 2 nS, based on the experimental observations of the dynamics of the input conductance.

The dynamics of the transient and persistent activity-dependent conductances are determined by the variables $G_{trans}\left(t\right)$ and $G_{pers}\left(t\right)$, respectively:

(16) $$\frac{d\;G_{trans}}{dt}=r_{trans}\;\nu \left(V\right)\;\chi^{syn}\left(t\right)\;\left(1-G_{trans}\right)-\frac{G_{trans}}{\tau_{tr}}$$

(17) $$\frac{d\;G_{pers}\;}{dt}=r_{pers}\;\nu \left(V\right)\;\chi^{syn}\left(t\right)\;\left(1-G_{pers}\right)-\frac{G_{pers}}{\tau_{pers}}$$

The function $W\left(t\right)$, which is present in Equations (14) and (15), sets the timing of blockade, i.e., the time window for the activation of additional conductances:

(18) $$W\left(t\right)=\frac{1}{1+\exp\left[-t+t_{start}/10,000\right]}\left(1-\frac{1}{1+\exp\left[-t+t_{end}/10,000\right]}\right)$$

Thus the activity-dependent conductances gradually increase as time approaches $t_{start}$= 120,000 ms and slowly deactivate as time approaches $t_{end}$ = 480,000 ms. Such smooth time-dependent block reflects a slow washing-in of the drug and helps to avoid abnormal patterns of epileptiform activity, which might take place if a step function is applied.

The dynamics of the extracellular potassium ion concentration ${\left[K^{+}\right]}_{o}\left(t\right)$ and the intracellular sodium ion concentration ${\left[Na^{+}\right]}_{i}\left(t\right)$ are as follows:

(19) $$\frac{d\;{\left[K^{+}\right]}_{o}\;}{dt}=r_{K}\;\nu \left(V\right)-\frac{2\;I_{pump}\left(t\right)}{F\;v}+\frac{2.5-{\left[K^{+}\right]}_{o}}{\tau_{K}}$$

(20) $$\frac{d\;{\left[Na^{+}\right]}_{i}\;}{dt}=r_{Na}\;\nu \left(V\right)-\frac{3\;I_{pump}\left(t\right)}{\gamma \;F\;v}+\frac{10-{\left[Na^{+}\right]}_{i}}{\tau_{Na}}$$

where the concentrations are in mM. The parameter γ represented the ratio of intracellular to extracellular volumes and was set equal to 5, which is close to the value given in [79]. F is the Faraday constant (96,485 C/mol), v represents the extracellular space volume per one neuron (300 µm^3^).

The Equations (16), (17), (19), (20) states that the firing of the main population increases $G_{pers}\left(t\right)$ and $G_{trans}\left(t\right)$, as well as ${\left[K^{+}\right]}_{o}\left(t\right)$ and ${\left[Na^{+}\right]}_{i}\left(t\right)$. When the population does not fire, these variables relax to their initial values. Fitting of the dynamics of activity-dependent conductances and ionic concentrations to our experimental observations gave: $r_{trans}$= 0.03 ms^−1^, $\tau_{tr}$ = 920 ms, $r_{pers}$ = 0.00065 ms^−1^, $\tau_{pers}\;$= 35,000 ms, $r_{K}$ = 0.0017 mM/ms, $\tau_{K}$ = 7500 ms, $r_{Na}$ = 0.0016 mM/ms, and $\tau_{Na}$ = 52,000 ms.

The membrane voltage of both populations is also affected by the Na^+^/K^+^ pump current, which is taken from [80] in the form

(21) $$I_{pump}\left(t\right)=\frac{I_{pump,MAX}}{\left(1+\exp\left(3.5-{\left[K^{+}\right]}_{o}\right)\right)\left(1+\exp\left[\left(25-{\left[Na^{+}\right]}_{i}\right)/3\right]\right)}$$

where $I_{pump,MAX}$ represents the maximal current set equal to 23 pA.

The other parameters were: C = 100 pF, $g_{Na,leak}$ = 0.4 nS, $g_{Cl,leak}$= 0.7 nS, $g_{K,leak}$= 1.9 nS, ${\left[K^{+}\right]}_{i}$ = 140 mM, ${\left[Na^{+}\right]}_{o}$= 151 mM, ${\left[Cl^{-}\right]}_{i}$= 10 mM, ${\left[Cl^{-}\right]}_{o}$ = 133 mM.

The initial conditions for the eqs. 2–5, 12–13, 16–17, 19–20 were as follows:

$V\left(0\right)$= −70.4 mV, $V_{Noisy}\left(0\right)$ = −70.4 mV, $G_{noise}\left(0\right)$= 0, $T_{syn}\left(0\right)$ = 0, $\chi^{syn}\left(0\right)$ = 1, $\chi^{NMDA}\left(0\right)$ = 1, $G_{trans}\left(0\right)$ = 0, $G_{pers}\left(0\right)$= 0, ${\left[K^{+}\right]}_{o}\left(0\right)$ = 2.5 mM, ${\left[Na^{+}\right]}_{i}\left(0\right)\;$= 10 mM

The system of Equations (2)–(5), (12)–(13), (16)–(17), (19)–(20) with the listed above set of initial conditions was solved numerically using the Wolfram Mathematica 12 software (the code is available at the following site https://yadi.sk/d/9JtcOlNh7weWgg, last access date 10 November 2021).

The total input conductance ($G_{input}$) in the model, which is reported in the results section, was calculated as a sum of the leak, transient and persistent conductances:

(22) $$G_{input}=g_{K,leak}+g_{Na,leak}+g_{Cl,leak}\;+\left(g_{trans}\;f_{trans}\left(V\right)G_{trans}\left(t\right)+g_{pers}\;G_{pers}\left(t\right)\right)\;W\left(t\right)$$

## Figures and Tables

**Figure A1:**
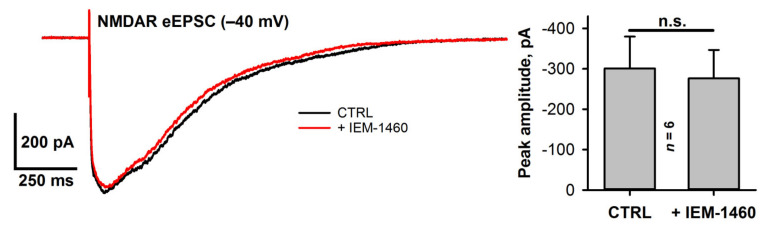
The effect of CP-AMPAR blocker IEM-1460 on NMDAR-mediated currents. NMDAR mediated eEPSC is not affected by IEM-1460. Left—representative examples of the averaged eEPSC before and after IEM-1460 application. Right—the peak amplitude of NMDAR mediated eEPSC did not change following IEM-1460 application (paired *t*-test, *p* = 0.2).

## Data Availability

The data presented in this study are available on request from the corresponding author.

## Appendix A

It was previously reported that IEM-1460 induces a flickering block of the NMDAR ion channel [81]. The ion channel of the NMDARs is several times more permeable to Ca^2+^ than that of CP-AMPARs [82], indicating that changes to NMDAR activation might be responsible for the reduced Ca^2+^ entry and the consequent decrease of the input conductance. In order to investigate whether the nonselective blockade of NMDARs by IEM-1460 can affect the results of the current study, we evaluated the effect of this drug on NMDAR-mediated EPSCs (Figure A1). To mimic the conditions during the epileptiform activity in the slice, the NMDAR-mediated EPSCs were recorded at −40 mV in the low Mg^2+^ containing solution. The bath application of IEM-1460 failed to induce any significant block of NMDAR-mediated evoked currents, indicating that the block of NMDARs is not responsible for the observed decrease of the Ca^2+^ sensitive conductance.
